# ID 73 - Eficácia do Uso da Escopolamina para Redução da Sialorreia em Crianças com Doenças Neurológicas

**DOI:** 10.5327/2237-9622.2025.v34s1.73

**Published:** 2025-11-25

**Authors:** Iara Beatriz Andrade de Sousa Algeri, Ellen Daiane Biavatti de Oliveira, Ricardo Fernandes, Francisco Rodrigues Martins, Rita De Cássia Dorácio Mendes, Rafaele Carla Pivetta Araújo, Dayse Sanches Guimaraes Paião, Rodrigo Alexandre Teixeira

**Keywords:** anticolinérgico, hipersalivação, uso *off-label*, tecnologia em saúde

## Abstract

**Introdução:**

O tratamento da sialorreia abrange o uso da toxina botulínica, tratamento cirúrgico e farmacológico (anticolinérgicos). O objetivo deste estudo foi analisar se a escopolamina (medicamento anticolinérgico) é eficaz para reduzir a sialorreia em crianças com doenças neurológicas (uso) em ambiente hospitalar.

**Método:**

Realizou-se uma revisão sistemática de ensaios clínicos quase-randomizados e controlados com buscas nas bases de dados: MEDLINE (PubMed); Embase e LILACS, via BVS e no Clinical Trials. Foram recuperadas 117 referências, inseridas no aplicativo Rayyan. Destas, duas referências foram incluídas para análise na revisão. As etapas de seleção foram executadas por dois investigadores independentes e o consenso por um terceiro avaliador. O desfecho analisado foi o número de pacientes que reduziram a sialorreia quando submetidos ao tratamento com escopolamina. A avaliação do risco de viés utilizou-se a ferramenta RoBins-I, para o desfecho analisado, e a certeza no conjunto final da evidência foi avaliada utilizando a abordagem GRADE.

**Resultados:**

Foram inclusos dois ensaios clínicos controlados (Jongerius et al., 2004a; Jongerius ., 2004b) que analisaram respectivamente 45 e 39 participantes. A partir da análise dos estudos observou-se que eles são oriundos de uma mesma pesquisa, com a mesma população, porém com análise de desfechos diferentes. Ambos compararam a toxina botulínica tipo A (BoNT) com adesivo transdérmico de escopolamina de 1,5 mg. No primeiro, foi avaliado a redução da salivação utilizando usando o Drooling Quotient (DQ). A análise do DQ demonstrou uma taxa de resposta de 53% dos pacientes à escopolamina, e 48,7% até 24 semanas após as injeções de BoNT. No segundo estudo foram avaliadas as taxas de fluxo salivar de todas as glândulas principais, obtidas no início do estudo e comparadas com as medições durante as intervenções. Comparado com a linha de base, a diminuição média no fluxo submandibular foi de 25% com a escopolamina e 42% após as injeções de BoNT. Em relação aos efeitos colaterais entre aqueles que desistiram de ambas as pesquisas (n=6), a maioria (n=4) estava sob uso de escopolamina. No primeiro estudo, 71,1% dos pacientes que apresentaram efeitos colaterais moderados a graves estavam no grupo que recebeu escopolamina. Apenas efeitos colaterais não graves e incidentais foram relatados após as injeções de BoNT. O risco de viés geral dos estudos foi grave, relacionado ao viés de confusão.

**Conclusão:**

A certeza no conjunto final da evidência dos desfechos primários foi considerada muito baixa, uma vez que a confiança na estimativa de efeito é muito limitada e a estimativa de efeito é incerta. A magnitude e a direção do efeito prevista dos vieses nesses estudos são imprevisíveis, pois os fatores confundidores não medidos e desconsiderados nas análises estatísticas poderiam tanto favorecer a intervenção (BoNT) quanto o comparador (escopolamina). No entanto, a maioria dos que desistiram de ambas as pesquisas estava sob uso de escopolamina, justamente devido aos importantes eventos adversos. Assim, sugere-se que a conduta clínica no tratamento da sialorreia seja pautado na melhor evidência científica. Para tal, é fundamental analisar estudos sobre a eficácia e a segurança de outras tecnologias, como a atropina, que possui baixo custo unitário, viabilidade de implantação e uma maior segurança, devido à ocorrência de menores efeitos colaterais, quando comparada a outas drogas anticolinérgicas.

